# Epstein-Barr Virus (Infectious Disease and Therapy)

**DOI:** 10.3201/eid1210.060778

**Published:** 2006-10

**Authors:** Joseph Pagano

**Affiliations:** * University of North Carolina, Chapel Hill, North Carolina, USA

**Keywords:** Epstein-Barr Virus, Alex Tselis, Book Review, review

Epstein-Barr virus (EBV) was the first recognized human tumor virus, but it is not the causative agent for the tumor in which it was discovered, Burkitt lymphoma. Common to all Burkitt lymphomas, endemic or sporadic, are distinctive chromosomal translocations that reactivate expression of the *c-myc* protooncogene and comprise the primary oncogenic mechanism.

EBV is at least a contributory cofactor in endemic Burkitt lymphoma, but the virus is detected in <20% of sporadic cases in the United States. EBV does cause infectious mononucleosis, hairy leukoplakia, and B-lymphoproliferative neoplasms in immunocompromised persons. In addition, the early and utterly consistent presence of monoclonal EBV episomes in nasopharyngeal carcinoma worldwide suggests a crucial role for the virus in that neoplasm. While tantalizing, associations with other diseases, well reviewed in this volume, are inconsistent and suggest that the virus may have another role beyond the etiologic, namely, by affecting the phenotype of already existing tumor cells and possibly propelling tumor progression.

This book is assembled mostly from a clinical perspective, and useful chapters on several of the EBV diseases bring together information not easily found elsewhere. Well-informed chapters on the virology and epidemiology of EBV infection are also included. One of the editors (whose list of milestones displays the clinical emphasis of the book) has provided a nice historical summary.

As is usual with such compilations, the editors leave it to the contributors to speak for themselves, and the quality of the chapters is uneven. Some fall short in citation of primary sources or favor the author's view rather than one that weighs all the evidence. Withal it is a useful book, and having the less often discussed associations such as T-cell lymphomas and leiomyosarcomas assigned a place alongside authoritative chapters on the classic associations, nasopharyngeal carcinoma and Burkitt lymphoma, is convenient. The volume ends with a chapter on an EBV vaccine, which remains elusive after many years. In contrast, the penultimate chapter includes a brief summary of some successes with adoptive immunotherapy for posttransplant lymphoproliferative disease, which is generally refractory to conventional treatment. This volume is worth having for the cross-section of knowledge and developments in the EBV field it presents.

